# Effects of Dehydration and Rehydration on Cognitive Performance and Mood among Male College Students in Cangzhou, China: A Self-Controlled Trial

**DOI:** 10.3390/ijerph16111891

**Published:** 2019-05-29

**Authors:** Na Zhang, Song M. Du, Jian F. Zhang, Guan S. Ma

**Affiliations:** 1 Department of Nutrition and Food Hygiene, School of Public Health, Peking University, Beijing 100191, China; zhangna@bjmu.edu.cn (N.Z.); zjf@bjmu.edu.cn (J.F.Z.); 2 Laboratory of Toxicological Research and Risk Assessment for Food Safety, Peking University, Beijing 100191, China; 3 Chinese Nutrition Society, Room 1405, Broadcasting Mansion, Beijing 100029, China; dusm9709@126.com

**Keywords:** water, hydration, dehydration, cognitive performance, mood

## Abstract

Water accounts for 75% of brain mass. Associations may exist between hydration and cognitive performance. The objective of this study was to investigate the effects of dehydration and rehydration on cognitive performance and mood. In this self-control trial, 12 men were recruited from a medical college in Cangzhou, China. After 12 h of overnight fasting, the participants took baseline tests at 8:00 AM on day 2. First morning urine and blood osmolality were analyzed to determine hydration state. Height, weight, and blood pressure were measured following standardized procedures. A visual analog scale for the subjective sensation of thirst was applied, and a profile of mood states questionnaire was applied. Tests were conducted for cognitive performance, including a test of digit span forward and backward, digit-symbol substitutions, dose-work, and stroop effects. Participants were required not to drink water for 36 h but were given three meals on day 3. On day 4, the same indexes were tested as a baseline test. At 8:30 AM, participants drank 1500 mL of purified water over 15 min. After a 1 h interval, the same measurements were performed. Compared with baseline test results, during the dehydration test, participants had lower scores of vigor (11.9 vs. 8.8, %, *p* = 0.007) and esteem-related affect (8.2 vs. 5.7, %, *p* = 0.006), lower total scores of digit span (14.3 vs. 13.3, %, *p* = 0.004), and higher error rates for dose-work (0.01 vs. 0.16, %, *p* = 0.005). Compared with the dehydration test scores, rehydration test scores showed that fatigue (4.3 vs. 2.1, %, *p* = 0.005) and total mood disturbance (TMD) (99.0 vs. 90.2, %, *p* = 0.008) improved, and scores of forward, backward, and total digit span increased (7.7 vs. 8.6, *p* = 0.014; 5.7 vs. 1.2, *p* = 0.019; 13.3 vs. 15.4, *p* = 0.001). Increases were also noted in correct number of digit symbol substitutions, reading speed, and mental work ability (70.8 vs. 75.4, *p* < 0.001; 339.3 vs. 486.4, n/min, *p* < 0.001; 356.1 vs. 450.2, *p* < 0.001), and reaction time decreased (30.2 vs. 28.7, s, *p* = 0.002). As a conclusion, dehydration had negative effects on vigor, esteem-related affect, short-term memory, and attention. Rehydration after water supplementation alleviated fatigue and improved TMD, short-term memory, attention, and reaction.

## 1. Introduction

Water is essential for the survival and development of life. The functions of water include its participation in metabolism, modulation of normal osmotic pressure, maintenance of electrolyte balance, and regulation of body temperature. Both excessive and insufficient water intake have negative effects on health [1,2]. Dehydration, which refers to a deficiency in total body water, can impair a person’s ability to engage in physical activities and increases the risk of urinary system and cardiovascular system diseases [3,4,5]. The brain regulates cognitive performance and mood, and water accounts for approximately 75% of brain mass [6]. Associations may exist between hydration states and cognitive performance and mood. 

A few studies have explored the effects of hydration on cognitive performance and mood. Some results have supported the hypothesis that cognitive performance and mood could be impaired by dehydration and improved by rehydration. Fadda et al. investigated the effects of water supplementation on the cognitive performance and subjective mood states of school children in a hot climate, revealing negative effects of dehydration on short-term memory and beneficial effects of water supplementation [7]. Armstrong et al. reported that dehydration elicited by intermittent moderate exercise without hyperthermia increased the perception of task difficulty and resulted in mood deterioration among 25 female participants [8]. Ganio et al. showed that mild dehydration without hyperthermia impaired vigilance and working memory and increased anxiety and fatigue among 26 men [9]. The studies of Cian, Lieberman, and D’anci et al. have also supported the view that hydration state could influence cognitive performance [10,11,12,13]. However, studies have obtained inconsistent conclusions. Wittbrodt et al. showed that rehydration after water supplementation effectively mitigated physiological strain induced by mild dehydration following exercise-heat stress, but mild dehydration had no adverse effects on cognitive performance among 12 recreational athletes [14]. A randomized trial conducted by Trinies et al. of primary school pupils in Zambia also demonstrated that provision of water or hydration level did not affect cognitive performance [15]. Some other studies have demonstrated that dehydration induced by heat stress and exercise did not impair cognitive performance [16,17,18,19]. In these studies, heat stress, physical activities, diuretics, or a combination of these, were used instead of water deprivation. In response to physical activities, brain-derived neurotrophic factor increases, brain glucose is elevated, and oxygen uptake and lactate uptake increase in the temporoparietal cortex, occipital cortex, premotor cortex, and cerebellar vermis. These responses may also affect cognitive performance and mood as confounding factors [20,21,22]. Heat stress may impair cognitive performance and mood due to the associated unpleasant sensation and increase in stress hormones [23]. Thus, it is necessary to conduct hydration-related studies using water deprivation and water supplementation under free-living conditions to explore the effects of progressive moderate dehydration and acute rehydration on cognitive performance and mood. 

In China, few hydration-related studies have been conducted. In one survey of 1483 adults aged 18–60 years from four provincial capital cities in 2009, approximately 32% of the participants reported that they drank less water than the amount recommended by the Chinese Nutrition Society in 2006 (1200 mL/day). Furthermore, 71.6% of participants had no knowledge of these recommendations [24]. Based on data from that water intakes survey, adequate water intake guidelines were reproposed by the Chinese Nutrition Society in 2013. The recommended adequate water intake levels for male and female adults were 1700 and 1500 mL, respectively. Another survey among young men in 2015 concluded that almost three-quarters of participants did not achieve the recommended amount of fluid intake (1700 mL) and a quarter were dehydrated [25]. Thus, conducting hydration-related studies is urgently required to emphasize the importance of hydration based on scientific evidence. However, few hydration-related health studies have been conducted, especially from the perspective of cognitive performance.

The objectives of this study involving young adults in China were first, to examine the effects of slow, progressive dehydration after 36 h of water deprivation on cognitive performance and mood, and second, to evaluate the effects of rehydration with water supplementation. 

## 2. Materials and Methods

### 2.1. Participant Recruitment

Twelve healthy men were recruited on the principle of voluntariness from freshman and sophomore years in one college in Cangzhou, Hebei Province, China. 

Inclusion criteria: Healthy male college students aged between 18 and 25 years were included. Exclusion criteria: Participants were excluded due to habitual high alcohol consumption (>20 g/day), tobacco use, vigorous-intensity physical activity habits (>6 METs), and cognitive disorders and other diseases [26]. 

### 2.2. Ethics

The study protocol and instruments were reviewed and approved by the Ethical Review Committee of the Chinese Nutrition Society (No.CNS-2015-001). The study was conducted according to the guidelines of the Declaration of Helsinki. Prior to the study, all participants read and signed informed consent forms.

### 2.3. Study Design 

**Figure 1:**
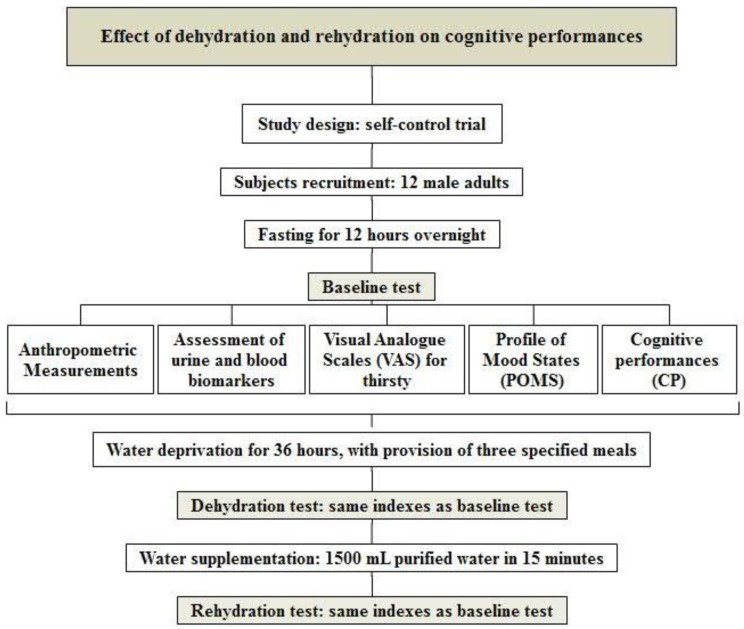
Flowchart of the study.

A self-control trial was designed and conducted. The flowchart of the study is shown in Figure 1.

In this study, the method of water deprivation used was inducing dehydration, whereas water supplementation was used to induce rehydration. Cognitive performance, mood, and thirst were measured three times: during the baseline test after fasting for 12 h, during the dehydration test after water deprivation for 36 h, and during the rehydration test after water supplementation.

### 2.4. Study Procedure 

**Figure 2:**
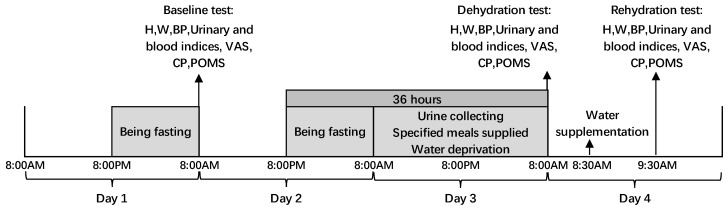
Procedure of the study. Note: H (height); W (weight); BP (blood pressure); VAS (visual analogue scales); CP (cognitive performance); POMS (profile of mood states).

The study procedure is shown in Figure 2.

*Day 1*: All participants were asked to fast from 8:00 PM. They were asked to try to sleep before 11 PM in the evening and to not urinate before the next morning so that first morning urine samples could be collected.

*Day 2*: All participants arrived at the designated location for the baseline test at 8:00 AM. The first morning urine samples were collected by participants themselves in sterile, disposable urine sample cups. The urine osmolality of participants was tested immediately by professional laboratory technicians at a hospital. Venous blood samples from the elbow were collected to test blood osmolality and blood glucose. Anthropometric measurements of height, weight, and blood pressure were also conducted. Then, a visual analog scale (VAS) for thirst, a profile of mood state (POMS) questionnaire, and a cognitive performance test were finished. After the baseline test, participants could drink water and have meals. Then, from 8:00 PM on day 2 to 8:00 AM on day 3, all participants were asked to fast, forgoing intake of both food and beverages.

*Day 3*: From 8:00 AM on day 3 to 8:00 AM on day 4, all participants were asked not to drink anything, including soup. They were, however, provided with solid foods for three meals. Breakfast was supplied at 7:00AM, lunch was given at 12:00 AM, and participants were supplied with dinner at 5:30 PM. No other food was allowed to be eaten except those three meals. All foods for the three meals eaten by participants were recorded and weighed. Fluid intake from food was determined with duplicate portion method according to the *National Food Safety Standard GB 5009.3-2010: Determination of Moisture in Foods*. Urine samples were collected to evaluate the amount of urine output over 24 h and to test urine osmolality. Participants were asked to try to sleep before 11 PM in the evening and not to urinate after going to bed until they got up the following morning; again, this was advised so that first urine samples could be collected.

*Day 4:* All participants arrived at the same designated location for the dehydration test at 8:00 AM. Dehydration test steps were the same as the baseline test (see the description of day 2). At 8:30 AM, participants were asked to drink 1500 mL of purified water during 8:30 AM to 8:45 AM (drinking 500 mL in each 5 min period). During an interval of 1 h, participants were asked to rest without engaging in physical activities. At 9:30 AM, the rehydration test was conducted as the steps of baseline test (see the description on day 2).

During the entire study period, all participants were asked not to engage in high-intensity physical activities. Anyone who failed to meet any of the abovementioned requirements were required to let the investigators know. 

### 2.5. Definition

Dehydration: Dehydration occurs if water intake is insufficient to replace free water loss [27]. The criterion of dehydration is a urine osmolality value of >800 mOsm/kg [28]. The criterion of optimal hydration is a urine osmolality value of ≤500 mOsm/kg [29].

Void volume and number: Random spot urine samples were recorded and collected starting with the second voiding of the day and ending with the first voiding the following morning. Void volume refers to the sum of the volume of each random spot urine sample. Void number refers to the total number of urinations during this meantime.

### 2.6. Assessment of Water Intake from Foods

All foods consumed by the participants on day 3 were weighted using a desktop electronic scale (YP20001; SPC; Shanghai, China). Each food sample was also collected separately using a duplicate portion method. Food samples were stored in refrigerators at 4 °C and sent to a laboratory to be measured within 36 hours. Water content of food was measured according to National Food Safety Standard GB 5009.3-2010 *Determination of water in Food* by a laboratory analyst at the Beijing Institute of Nutritional Resources. The error between the two parallel food samples was no more than 5%. 

### 2.7. Temperature and Humidity

The indoor and outdoor temperature and humidity at the study site were recorded using a temperature hygrometer at 10:00 AM, 2:00 PM, and 8:00 PM during the study period. The study site was a classroom in the college. Participants spend approximately 8 h in the classroom each day. The dormitories of participants were within 1 km of the classroom. As such the temperature and humidity at the study site were representative of the environments in which the participants live and study.

### 2.8. Anthropometric Measurements

Height and weight were measured twice by trained investigators using a height-weight meter (HDM-300; Huajun, Zhejiang, China) while participants were in bare feet and wearing light clothing, during 8:00 AM to 8:30 AM on the water supplementation day. Height (H) was measured twice to the nearest 0.1 cm and weight (W) was measured twice to the nearest 0.1 kg.

BMI (body mass index, kg/ m^2^) = weight (kg) / height squared (m^2^) (1)

Blood pressure (BP) was measured twice by a nurse using an electronic sphygmomanometer (HEM-7051; Omrom, Dalian, Liaoning, China) during the same standardized time. Two measurements were taken after 2 min intervals, and measurements were to the nearest 2 mmHg. 

### 2.9. Assessment of Urine Biomarkers

Urine samples were collected in self-designed, disposable flexible, plastic bags by participants. Urine samples were stored at 4 °C prior to assessment and were analyzed within 2 h. Urine volume was measured to the nearest 0.1 kg using a desktop electronic scale (YP20001; SPC; Shanghai, China). The urine osmolality of each urine sample was tested by a laboratorian with an osmotic pressure molar concentration meter (SMC 30C; Tianhe, Tianjin, China) using the freezing-point method. 

### 2.10. Assessment of Blood Biomarkers

Venous blood from the elbow was also collected to determine blood osmolality and blood glucose. Blood osmolality were tested by a laboratorian using the same equipment and method used for testing blood osmolality. Blood glucose levels were tested by a laboratorian with an automatic biochemical analyzer (Cobas C501; Roche, Basel, Switzerland) using the ion-selective electrode potentiometer method. 

### 2.11. VAS for Subjective Thirst Sensation

In this study, the subjective sensation of thirst was measured using a VAS. On the self-rated 10 cm horizontal line the of the VAS, the labels “not at all” and “extremely” were anchored at the beginning and end, respectively, to quantitatively measure subjective the aforementioned sensation [30]. According to their feelings at the time of testing, participants draw a vertical line on this horizontal line. The range of scores for thirst was from 0 to 10 [26]. 

### 2.12. POMS

This self-rating mood questionnaire consisted of seven subscales and 40 adjectives that measured tension, depression, fatigue, vigor, confusion, anger, esteem-related affect, and total mood disturbance. Total mood disturbance (TMD) is a global estimate of affective state [31]. All scores for each adjective were determined using a 5-point scale (0, “not at all,” 1, “a little,” 2, “moderately,” 3, “quite a bit,” and 4, “extremely” [26,31]. Participants were instructed to choose one point for each adjective that conformed to their own situation. 

The score for TMD was obtained as follows: score of (tension + depression + anger + fatigue + confusion) − score of (vigor + esteem-related affect) + 100.

### 2.13. Cognitive Performance

Digit span forward and backward: In this test, a list of random numbers was read aloud by trained investigators at a uniform rate of one number per second. Then, participants recalled the digit sequence in forward or backward order. This test was used to assess short-term memory. 

The total score was obtained as follows: score of forward digit span + score of backward digit span [26,32].

Digit symbol substitution test: This test consisted of nine digit-symbol pairs followed by a list of digits (total 90) (for example, 1/_, 2/⊥ ... 7/Λ, 8/X, 9/=). Participants wrote down the corresponding symbol under each digit as fast as possible within 90 s. The number of correct symbols was calculated for scoring [26,33]. This test was used to assess participants’ abilities related to digital decoding, visual memory, visual attention, and operating speed.

Dose-work test: An Aventura Karimov table was used to evaluate the quality and quantity of dose-work [34]. This test was used to assess sustained attention. 

Reading speed (n/min) = number of already-read letters (*n*)/2 (min).

Error rate (%) = number of errors (*n*)/number of already-read letters (*n*) × 100%.

Mental work ability index (IMC) = number of already-read letters (*n*)/2 (min) × (number of letters that had to be crossed − number of errors)/number of letters that had to be crossed [26].

Stroop effect test: This was used to test reaction ability. Words were displayed in a color different from the color it actually names (e.g., the word "green" was printed in blue ink instead of green ink). Then, participants were asked to read the actual color the printed word. The number of correct symbols and the reaction time were measured for scoring [35,36].

### 2.14. Statistical Analysis

SAS version 9.2 (SAS Institute Inc., Cary, NC, USA) was used for statistical analyses. Quantitative parameters for participants were presented as mean ± standard deviation; classified variables (hydration state) were presented as numbers and percentages. Quantitative parameters were compared using a one-way analysis of variance (ANOVA) with replicate measures among the baseline test, dehydration test and rehydration test; statistical significance was set at 0.05 (*p* < 0.05, 2-tailed) with 95% confidence intervals (95% CIs). Dunnett’s method was used when multiple comparisons were conducted, and the level of significance was set at 0.025 (*p* < 0.025, 2-tailed) with 95% confidence intervals (95% CI). Binary classification data were compared using the chi-squared test.

## 3. Results

### 3.1. Characteristics of the Participants 

A total of 12 male participants were recruited and completed the study (100% completion rate). The average age of participants was 20.8 years old (range: 19.2–23.7 years). The height, weight, BMI, and blood pressure measurements obtained during baseline, dehydration, and rehydration tests are shown in Table 1. No significant differences were observed in these anthropometric measurements over these three tests.

### 3.2. Temperature and Humidity

The average temperature during days 1 to 4 at the study site was 16.2 °C inside the classroom and 20.4 °C outside, whereas the average humidity was 32% inside the classroom and 33% outside. 

### 3.3. Fluid from Food, 24 h Urine Volume, Void Number, and Urine Osmolality on Day 3

**Figure 3:**
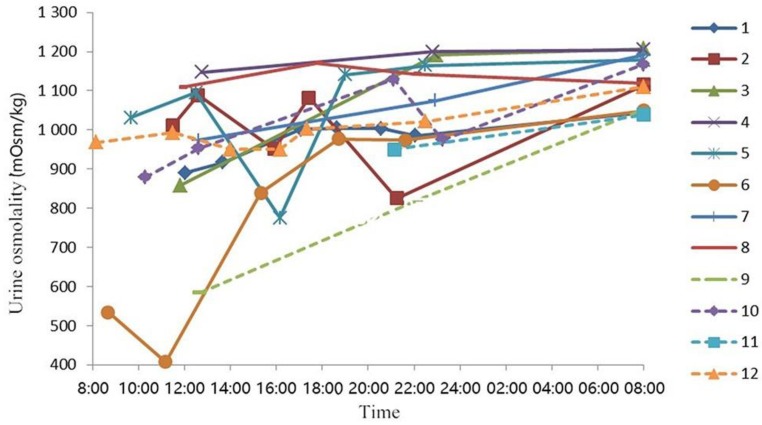
Changing trend of urine osmolality for each subject on day 3.

On day 3, the average total fluid volume obtained from food was 939 ± 146 mL, 24 h urine volume was 799 ± 145 mL, and the number of voids was 5 ± 2. These data are presented in Online Resources Table S1 (in supplementary materials). The average urine osmolality was 1004 ± 163 mOsm/kg. Changing trends of urine osmolality are shown in Figure 3, and the volume and osmolality for each spot urine sample are shown in Online Resources Table S2. Among the 12 participants, 9 (75%) were in a state of dehydration for the whole of day 3 (Table S3).

### 3.4. Hydration State, Thirst Perception, Related Urine, and Blood Biomarkers

No significant differences were observed in blood osmolality, blood glucose, urine osmolality, thirst, and hydration over all test periods.

Compared with baseline test measurements, urine osmolality and thirst scores during the dehydration test were statistically higher (*F* = 32.8, *p* < 0.001; *F* = 19.62, *p* = 0.001), and more participants were in a dehydration state than were not (*χ*^2^ = 8.000, *p* = 0.005). Compared with dehydration test measurements, during the rehydration test, participants had lower blood osmolality, urine osmolality, and thirst scores (*F* = 23.31, *p* = 0.001; *F* = 100.95, *p* < 0.001; *F* = 27.64, *p* < 0.001), and fewer were in a dehydration state than were not (*χ*^2^ = 20.308, *p* < 0.001; Table 2).

### 3.5. Effects of Dehydration and Rehydration on POMS

Moods related to fatigue, vigor, esteem-related affect, and TMD were significantly different during the baseline, dehydration, and rehydration tests.

Compared with baseline test scores, scores of vigor and esteem-related affect of participants during the dehydration test were significantly lower (*F* = 11.06, *p* = 0.007; *F* = 11.30, *p* = 0.006). Scores of fatigue and TMD of participants during the rehydration test were significantly lower than those obtained during the dehydration test (*F* = 12.32, *p* = 0.005; *F* = 10.28, *p* = 0.008; Table 3).

### 3.6. Effects of Dehydration and Rehydration on Cognitive Performance 

Significant differences were noted in forward, backward, and total scores of digit span, correct number of digit symbol substitutions, reading speed, error rate and IMC of dose-work, and reaction time during the stroop test during the baseline, dehydration, and rehydration tests.

During the dehydration test, participants had lower total scores of digit span and higher error rates of dose-work compared with their baseline test scores (*F* = 13.20, *p* = 0.004; *F* = 12.05, *p* = 0.005). Compared with their dehydration test scores, for the rehydration test, participants achieved higher forward, backward and total scores of digit span, more correct digit symbol substitutions, a faster reading speed and higher IMC of dose-work, and a shorter reaction time for the stroop test (*F* = 8.59, *p* = 0.014; *F* = 7.74, *p* = 0.019; *F* = 33.87, *p* = 0.001; *F* = 13.78, *p* = 0.003; *F* = 57.45, *p < 0.001*; *F* = 77.38, *p* < 0.001; *F* = 16.27, *p* = 0.002; Table 4).

## 4. Discussion

In the study, dehydration induced by water deprivation for 36 h had negative effects on vigor and esteem-related affect. It impaired the cognitive performance, such as that related to short-term memory and attention. Another study with 16 participants in this field showed that tiredness was increased and alertness was reduced after 24 h of water deprivation; in addition, reaction times were prolonged in women but shortened in men [37]. Similarly, during 24 h of voluntary water deprivation in one study of 10 participants, a significant deterioration was observed in cognitive performance, such as that related to solving time in psychological tests; however, self-estimated mood did not changed significantly [38]. In a study of athletes, it was revealed that mild dehydration after 12 h of water deprivation impaired cognitive-motor task performance, such as judgment of distance to a target [39]. Few cognitive performance studies related to dehydration have been conducted using water deprivation to induce dehydration. Most studies obtained similar results, revealing that dehydration impaired cognitive performance and mood; however, the mechanisms behind these effects have not been fully studied. A study demonstrated that water deprivation influences the transcription of many genes in the brain, such as the upregulation of clathrin, serum/glucocorticoid-regulated kinase, and protein kinase anchor protein 8-like; and the downregulation of janus kinase, microtubule interacting protein 1, and neuronal PAS domain protein 4 [40]. In some studies, MRI was conducted to explore these related mechanisms among participants in the acute stage of dehydration. It was revealed that the ventricular system expanded, with the most substantial change occurring in the left lateral ventricle in one study, which may induce the short-term changes in cognitive performance [41]. In another study, using a brain MRI scan, it was demonstrated that the lateral ventricle was enlarged and fronto-parietal blood-oxygen-level-dependent response was increased after acute dehydration induced by a thermal exercise protocol. These changes may be related to the complex mechanisms behind the brain’s response to dehydration.

As stated, a key concern of this study was whether rehydration after water supplementation could improve cognitive performance and mood. The results determined that sufficient water supplementation is an effective method of ensuring optimal hydration and improving cognitive performance and mood. A placebo-controlled crossover design study of 16 male participants showed that rehydration after dehydration induced by exercise attenuated alcohol-related impairment of cognitive functions [42]. In another study of 32 young adults and 30 older adults, the alertness and attentiveness of participants increased, their fatigue decreased, and their reaction ability improved after rehydration. Furthermore, one study confirmed the dose-response effects of water supplementation on cognitive performance in children and that visual attention can be enhanced by even a small intake of water (25 mL) [43]. For school children, it was revealed that focusing on drinking an adequate volume of water to retain optimal hydration throughout the day may be the key to enhancing cognitive performance. However, the results of some studies have been inconsistent [44,45]. A systematic review concluded that evidence for the benefits of water supplementation on cognitive performance is insufficient, with further confirmation required [46]. Different methods of inducing dehydration, degrees of dehydration, methods of conducting cognitive performance tests, and amounts of water supplementation may have caused these inconsistent conclusions. Thus, additional studies should be conducted to elucidate the effects of water supplementation on cognitive performance. The plausible mechanism should also be explored through the aspects of hormonal, neurochemical, and vascular functions in response to hydration status. 

The study has certain strengths and limitations. Only water deprivation was used to induce dehydration. A pure method of water deprivation and water supplementation in free-living conditions may be more helpful for exploring the effects of hydration on cognitive performance. Urine osmolality during the period of water deprivation was monitored to changes in hydration status and to verify the adherence of participants. The obtained urine osmolality trends suggested that the osmolality of most participants increased with the prolongation of water deprivation. Furthermore, nine participants (75%) were in a state of dehydration for the whole day. These reflect the favorable compliance of participants and the high quality of the study process. In terms of limitations, the effects of long-term water interventions and the mechanisms governing hydration and cognitive performance were not studied. Only male participants were recruited, thus gender differences were not studied. The conditions at each participant’ places were not recorded. Water loss through sweating and excretion of feces were not monitored in this study. 

## 5. Conclusions

Dehydration had negative effects on vigor, esteem-related affect, short-term memory, and attention. Rehydration after water supplementation improved fatigue, TMD, short-term memory, attention, and reaction. Our results confirm that drawing attention to the importance of water intake and hydration is necessary. Water-related health education should be provided effectively to advocate drinking adequate amounts of water and maintaining a state of optimal hydration.

## Figures and Tables

**Table 1:** Anthropometric measurements of subjects.

Anthropometric Measurements	Baseline Test	Dehydration Test	Rehydration Test	*F*	*p*
Height (cm)	176.0 ± 5.5	176.0 ± 5.5	176.0 ± 5.5	-	-
Weight (kg)	68.0 ± 10.9	67.2 ± 10.5	68.4 ± 10.3	0.04	0.963
BMI (kg/m^2^)	21.9 ± 3.0	21.6 ± 2.9	22.0 ± 2.8	0.05	0.948
Blood pressure					
Systolic pressure (mmHg)	114 ± 7	112 ± 8	116 ± 8	0.914	0.411
Diastolic pressure (mmHg)	75 ± 8	73 ± 7	75 ± 5	0.175	0.840

Note: *F*, statistics of variance analysis; *p* < 0.05 has statistically significance.

**Table 2:** Hydration state, thirsty, related urine and blood biomarkers of subjects.

Hydration State and Related Biomarkers	Baseline Test	Dehydration Test	Rehydration Test	*F*	*p*
Blood indices					
Osmolality (mOsm/kg)	304.6 ± 7.1	305.7 ± 6.4	295.3 ± 7.8 #	11.98	<0.001 ^†^
Glucose (mmol/L)	4.3 ± 0.3	4.5 ± 0.4	4.9 ± 0.2 #	11.67	<0.001 ^†^
Urine indices					
Osmolality (mOsm/kg)	803.2 ± 171.7 *	1123.3 ± 65.7	387.0 ± 268.3 #	49.13	<0.001 ^†^
Thirsty	3.3 ± 2.2 *	6.8 ± 2.6	1.9 ± 1.5 #	20.06	<0.001 ^†^
Hydration state					
Dehydration state	6 (50.0%) *	12 (100.0%)	1 (8.3%) #	34.58 ^Ф^	<0.001 ^†^
Optimal hydration state	0 (0.0%) *	0 (0.0%)	9 (75.0%) #		
Middle hydration state	6 (50.0%) *	0 (0.0%)	2 (16.7%) #		

Note: *, There were statistically significant differences between the baseline test and dehydration test, *p* < 0.025. #, There were statistically significant differences between the dehydration test and rehydration test, *p* < 0.025. ^†^, There were statistically significant differences between the three tests, *p* < 0.05. ^Ф^, the statistical value was χ^2^ of Chi-square.

**Table 3:** POMS of subjects.

POMS	Baseline Test	Dehydration Test	Rehydration Test	*F*	*p*
Tension	3.4 ± 2.5	3.0 ± 3.6	1.8 ± 2.7	2.21	0.134
Anger	0.9 ± 1.7	1.1 ± 1.6	0.1 ± 0.3	2.26	0.092
Fatigue	2.6 ± 2.0	4.3 ± 3.8	2.1 ± 3.0 #	3.93	0.035 ^†^
Depression	1.2 ± 1.6	2.2 ± 3.1	0.9 ± 1.6	2.03	0.155
Confusion	2.9 ± 2.2	2.8 ± 2.4	1.6 ± 1.9	2.33	0.136
Vigor	11.9 ± 2.5 *	8.8 ± 4.5	9.8 ± 4.3	5.52	0.011 ^†^
Esteem-related affect	8.2 ± 2.2 *	5.7 ± 2.3	6.5 ± 2.3	7.16	0.004 ^†^
TMD	90.9 ± 8.6	99.0 ± 17.0	90.2 ± 12.3 #	3.94	0.035 ^†^

Note: *, There was statistically significant differences between baseline test and dehydration test, *p* < 0.025. #, There were statistically significant differences between dehydration test and rehydration test, *p* < 0.025. ^†^, There were statistically significant differences between three tests, *p* < 0.05.

**Table 4:** Cognitive performance of subjects.

Cognitive Performance	Baseline Test	Dehydration Test	Rehydration Test	*F*	*p*
Digit span					
Forward score	7.9 ± 0.9	7.7 ± 0.8	8.6 ± 0.9 #	6.39	0.007 ^†^
Backward score	6.4 ± 1.3	5.7 ± 1.2	6.8 ± 1.6 #	5.01	0.016 ^†^
Total score	14.3 ± 1.8	13.3 ± 1.4	15.4 ± 2.2 #	15.83	<0.001 ^†^
Digit symbol substitution					
Correct number (*n*)	67.5 ± 6.0	70.8 ± 5.4	75.4 ± 4.6 #	14.93	<0.001 ^†^
Dose-work					
Reading speed (n/min)	339.9 ± 76.6 *	339.3 ± 69.8	486.4 ± 104.4 #	43.63	<0.001 ^†^
Error rate (%)	0.01 ± 0.04 *	0.16 ± 0.15	0.12 ± 0.11	5.40	0.012 ^†^
IMC	337.3 ± 75.9	356.1 ± 83.3	450.2 ± 88.4 #	33.73	<0.001 ^†^
Stroop					
Reaction time (s)	30.7 ± 2.6 *	30.2 ± 2.2	28.7 ± 1.7 #	10.05	<0.001 ^†^
Correct number (*n*)	23.3 ± 1.2	22.3 ± 1.9	23.2 ± 1.2	1.69	0.200

Note: *, There were statistically significant differences between the baseline test and dehydration test, *p* < 0.025. #, There were statistically significant differences between dehydration test and rehydration test, *p* < 0.025. ^†^, There were statistically significant differences between the three tests, *p* < 0.05.

## Supplementary Materials

The following are available online at https://www.mdpi.com/1660-4601/16/11/1891/s1, Table S1: Total fluid from food, 24 h urine volume, void number of subjects on day 3, Table S2: The osmolality and volume for each spot urine among subjects on day 3 [urine osmolality(mOsm/kg) /urine volume(mL)], Table S3: Hydration state of subjects on day 3 [void number/percentage].
