# Supplementary material for: Effects of Dehydration and Rehydration on Cognitive Performance and Mood among Male College Students in Cangzhou, China: A Self-Controlled Trial
Source: Int J Environ Res Public Health. 2019 May 29;16(11):1891. doi: 10.3390/ijerph16111891 (PMC6603652; doi:10.3390/ijerph16111891)
Supplement: Supplementary file 1 [file ijerph-16-01891-s001.pdf]

International Journal of
Environmental Research and Public Health

Supplementary Material

Effects of Dehydration and Rehydration on Cognitive Performance and Mood among Male College Students in Cangzhou, China: A Self-Controlled Trial

Na Zhang, Song M Du, Jian F Zhang and Guan S Ma *

Table S1. Total fluid from food, 24 h urine volume, void number of subjects on day 3.

Subjects ID	Total Fluid from Food (ml)	24 h Urine Volume (ml)	Void Number
1	1029.2	999.3	7
2	1051.4	746.3	6
3	911.7	832.3	3
4	958.6	744.5	3
5	1042.8	722.3	6
6	1130.4	1031.0	6
7	610.9	551.9	3
8	962.2	940.7	4
9	751.6	668.0	3
10	1043.6	882.5	5
11	936.0	661.5	2
12	839.2	811.0	7
Average±Standard Deviation	939.0 ± 145.7	799.3 ± 144.9	5 ± 2

Table S2. The osmolality and volume for each spot urine among subjects on day 3 (urine osmolality(mOsm/kg) /urine volume(mL)).

Subjects ID	The 1st Spot Urine	The 2nd Spot Urine	The 3rd Spot Urine	The 4th Spot Urine	The 5th Spot Urine	The 6th Spot Urine	The 7th Spot Urine
1	890/257.0	917/36.6	1001/71.2	1005/188.5	1003/107.0	985/132.0	1045/207.0
2	1012/229.8	1089/125.8	951/34.3	1082/137.5	825/61.9	1115/157.0	-/-
3	858/275.8	1191/187.2	1207/369.3	-/-	-/-	-/-	-/-
4	1147/519	1200/147.1	1205/78.4	-/-	-/-	-/-	-/-
5	1031/165.8	1095/107.8	775/117.4	1141/146.2	1164/118.9	1178/66.2	-/-
6	534/275.6	408/226.1	839/216.6	977/138.7	974/107.0	1049/67.0	-/-
7	974/274.4	1075/110.5	1192/167	-/-	-/-	-/-	-/-
8	1109/366.5	1171/199.5	1143/202.8	1120/171.9	-/-	-/-	-/-
9	584/216.5	820/278.7	1052/172.8	-/-	-/-	-/-	-/-
10	879/234.1	953/116.4	1129/93.0	975/232.0	1168/207.0	-/-	-/-

11	950/314.5	1039/347.0	-/-	-/-	-/-	-/-	-/-
12	968/211.0	993/59.9	950/113.9	950/93.7	1002/77.2	1022/97.9	1110/157.4

Note: Data were shown as urine osmolality (mOsm/kg) /urine volume(mL).

Table S3. Hydration state of subjects on day 3 (void number/percentage).

Subjects ID	Dehydration State	Optimal Hydration State	Middle Hydration State
1	7/100%	0/0%	0/0%
2	6/100%	0/0%	0/0%
3	3/100%	0/0%	0/0%
4	3/100%	0/0%	0/0%
5	5/83.3%	0/0%	1/16.7%
6	4/66.7%	1/16.7%	1/16.7%
7	3/100%	0/0%	0/0%
8	4/100%	0/0%	0/0%
9	2/66.7%	0/0%	1/33.3%
10	5/100%	0/0%	0/0%
11	2/100%	0/0%	0/0%
12	7/100%	0/0%	0/0%

Note: Data were shown as void number/percentage (%).

© 2019 by the authors. Submitted for possible open access publication under the terms and conditions of the Creative Commons Attribution (CC BY) license (http://creativecommons.org/licenses/by/4.0/).
